# Physical condition and stress levels during early development reflect feeding rates and predict pre- and post-fledging survival in a nearshore seabird

**DOI:** 10.1093/conphys/cow060

**Published:** 2016-12-09

**Authors:** Juliet S. Lamb, Kathleen M. O'Reilly, Patrick G. R. Jodice

**Affiliations:** 1 Department of Forestry and Environmental Conservation, and South Carolina Cooperative Fish and Wildlife Research Unit, G-27 Lehotsky Hall, Clemson University, Clemson, SC 29634, USA; 2 Department of Biology, University of Portland, 5000 N Willamette Boulevard, Portland, OR 97203, USA; 3 US Geological Survey South Carolina Cooperative Fish and Wildlife Research Unit, and Department of Forestry and Environmental Conservation, G-27 Lehotsky Hall, Clemson, SC 29634, USA

**Keywords:** Body condition, energy delivery, feather corticosterone, nest productivity, stressors

## Abstract

The effects of acute environmental stressors on reproduction in wildlife are often difficult to measure because of the labour and disturbance involved in collecting accurate reproductive data. Stress hormones represent a promising option for assessing the effects of environmental perturbations on altricial young; however, it is necessary first to establish how stress levels are affected by environmental conditions during development and whether elevated stress results in reduced survival and recruitment rates. In birds, the stress hormone corticosterone is deposited in feathers during the entire period of feather growth, making it an integrated measure of background stress levels during development. We tested the utility of feather corticosterone levels in 3- to 4-week-old nestling brown pelicans (*Pelecanus occidentalis*) for predicting survival rates at both the individual and colony levels. We also assessed the relationship of feather corticosterone to nestling body condition and rates of energy delivery to nestlings. Chicks with higher body condition and lower corticosterone levels were more likely to fledge and to be resighted after fledging, whereas those with lower body condition and higher corticosterone levels were less likely to fledge or be resighted after fledging. Feather corticosterone was also associated with intracolony differences in survival between ground and elevated nest sites. Colony-wide, mean feather corticosterone predicted nest productivity, chick survival and post-fledging dispersal more effectively than did body condition, although these relationships were strongest before fledglings dispersed away from the colony. Both reproductive success and nestling corticosterone were strongly related to nutritional conditions, particularly meal delivery rates. We conclude that feather corticosterone is a powerful predictor of reproductive success and could provide a useful metric for rapidly assessing the effects of changes in environmental conditions, provided pre-existing baseline variation is monitored and understood.

## Abstract (precis)

Snapshot nestling health measures can reveal reproductive effects of environmental stress while minimizing handling, disturbance and researcher effort. We tested two short-term measurements, body condition and feather corticosterone, as predictors of brown pelican nestling survival. Both measurements predicted nestling survival to fledge, and feather corticosterone also predicted post-fledging survival.

## Introduction

Impacts of acute or chronic environmental stressors on wildlife are typically quantified directly using mortality rates derived from carcass counts (Piatt *et al*., 1990; Burger, 1993) or multiyear census data (Wiens *et al*., 1996; Yaukey, 2012), which are then incorporated into demographic models to estimate the population-level effects of stressors (Haney *et al*., 2014). In addition to causing immediate mortality, however, stressors can also act sublethally through secondary pathways including reduced habitat quality (Cheng *et al*., 2009; Williams *et al*., 2010), compromised physical condition (Romero and Wikelski, 2001), physiological and genetic modifications (Møller and Mousseau, 2011) or increased susceptibility to existing threats, such as disease or environmental fluctuation (Balseiro *et al*., 2005; Whitehead, 2013). Many of these indirect and sublethal stressors subsequently impact demographic processes by reducing long-term survival or reproductive fitness in surviving individuals (Krebs and Burns, 1977; Peterson, 2001) but often are not explicitly or adequately addressed in demographic calculations and projections. Moreover, as reductions in adult condition and habitat suitability make it less likely for breeders to meet the energetic demands of territory defense, egg production, incubation and provisioning young, the breeding process itself is likely to compound impacts of environmental stress (Butler *et al*., 1988; Gannon and Willig, 1994). Indeed, demographic models that do not accurately incorporate secondary effects of environmental stressors on breeding success and recruitment cannot accurately predict or quantify the complex population-level impacts of environmental perturbations (Peterson *et al*., 2003; Haney *et al*., 2014).

Despite widespread understanding of the capacity of sublethal environmental stress to affect reproduction and recruitment negatively, it can be difficult to determine the most appropriate end points for measuring these effects (Smits and Fernie, 2013). In order for post-disturbance measurements to be informative, there must be a pre-existing understanding of the level of variation in reproductive parameters expected in baseline conditions (Teal and Howarth, 1984; Velando *et al*., 2005). Such data are not always available for species of interest prior to catastrophic events (Eppley, 1992). Moreover, the collection of reproductive data can be time and labour intensive and can involve researcher disturbance, which may make it difficult to implement rapidly in the wake of unexpected external change (Wiens *et al*., 1984). Snapshot measures of reproductive health, such as body condition index (BCI; e.g. Jakob *et al*., 1996; Benson *et al*., 2003), can be collected during a single visit and with minimal disturbance, allowing for rapid data collection across large areas after disturbance events. However, these measures have high short-term variability, and their relationship to demographic parameters of interest (e.g. reproductive success) must be evaluated in order to select appropriate metrics.

Stress hormone production offers a broadly applicable metric for assessing the impacts of environmental stressors on free-living wildlife populations (Romero and Wikelski, 2001). Corticosterone (CORT) is the principal glucocorticoid stress hormone in birds, rodents, reptiles and amphibians, and is frequently used as a measure of individual stress responses to environmental conditions and disturbance (e.g. Marra and Holberton, 1998; Kitaysky *et al*., 2001; Blas *et al*., 2005; Bonier *et al*., 2007; Almasi *et al*., 2009). Stress hormones are upregulated in response to perceived stressors, prompting short-term behavioural adjustments (e.g. aggression, submissiveness) and physiological modifications (e.g. immunosuppression; McEwen *et al*., 1997). Over time, however, chronic elevation in corticosterone levels in response to chronic stress may negatively affect organism health by altering immune function, growth rates, body condition and behaviour (Sapolsky *et al*., 2000). Corticosterone levels in adult individuals can be confounded by short-term energetic and behavioural differences (Angelier *et al*., 2007) and may change over life stages (Williams *et al*., 2008; Bonier *et al*., 2009). Within avian taxa, measuring corticosterone in altricial young controls for some of these influences, as their exposure to stress is localized and their range of behavioural responses restricted (Kitaysky *et al*., 2003; Eggert *et al*., 2010). Short-term exposure to acute stress during development can have positive effects, such as preparing individuals to respond more effectively to novel environments and unpredictable conditions later in life (Spencer *et al*., 2009; Zimmer *et al*., 2013). However, chronic elevation in stress levels is associated with severe developmental consequences, including reduced growth rates, impaired cognitive ability and elevated mortality in adulthood (e.g. Kitaysky *et al*., 2003; Müller *et al*., 2009; Butler *et al*., 2010; Monaghan *et al*., 2012). Therefore, differences in nestling corticosterone levels provide an initial indication of potential long-term differences in individual survival and reproductive performance, which ultimately drive population dynamics (Kitaysky *et al*., 2010).

While corticosterone levels in blood plasma can be elevated by short-term factors, such as stress resulting from capture (Love *et al*., 2003; Romero and Reed, 2005), corticosterone in avian feathers provides a more sustained record of stress levels over days or weeks (Bortolotti *et al*., 2008; Harms *et al*., 2010; Romero and Fairhurst, 2016). Feather corticosterone measurements allow for direct comparison of long-term nestling stress levels between different breeding habitats, where variations in nutrition, contamination, predation and parental attendance may affect chronic chick stress even if no outward physical differences are apparent (Bortolotti *et al*., 2009; Harms *et al*., 2010). Recent laboratory and field studies have demonstrated that chronic nutritional stress affects feather corticosterone levels in both captive and free-living seabirds (Patterson *et al*., 2015; Will *et al*., 2015), indicating that feather corticosterone is an appropriate metric for evaluating effects of stressors on chick development.

We assessed the utility of two snapshot nestling health measures, feather corticosterone (feather CORT) and BCI, for assessing reproductive success in the brown pelican (*Pelecanus occidentalis*), a nearshore seabird with altricial chicks that is frequently subjected to acute environmental stressors, including disturbance, oil exposure and hurricanes (Wilkinson *et al*., 1994). Body condition indices are based on nestling mass, which represents both long-term lipid reserves and short-term fluctuations (Labocha and Hayes, 2012), whereas feather CORT integrates levels of hormone deposition during the 2–3 week period of feather growth preceding feather collection. We assessed the relationship of feather CORT and BCI to survival probability of individual nestlings, as well as to correlative population-based measures of nutritional stress, colony-wide fledging success and post-fledging dispersal. Our predictions were as follows: (i) 3- to 4-week-old nestlings with higher BCI would have lower levels of feather CORT (and vice versa); (ii) the probability of individual nestlings surviving to fledge would increase with increasing body condition and decreasing corticosterone measured at 3–4 weeks of age; (iii) colony-wide fledging success would be highest at colonies with higher average body condition and lower feather corticosterone measured in 3- to 4-week-old chicks; and (iv) feather corticosterone would increase and BCI decrease with increasing nutritional stress, measured by lower rates of energy delivery to nestlings.

## Materials and methods

### Study species

The brown pelican is a large-bodied nearshore seabird that inhabits marine environments year-round (Shields, 2014). Brown pelicans feed on schooling fish by plunge-diving, and can carry large masses of fish in a single pouch-load while feeding nestlings. They nest in large offshore colonies that can number several thousand individuals. Nest elevation can vary widely depending on available habitat, from open ground to tree sites up to 10 m in elevation. Brown pelicans typically lay three sequentially hatching eggs, which require an incubation period of ~30 days, and raise one or two young. Although nestlings can fly at ~60 days, they generally do not leave the nesting colony until 70–90 days after hatching. Brown pelicans exhibit biparental care and feeding throughout the nesting period. At least one parent attends the nest at all times until chicks become mobile (~3–4 weeks), after which point parents are generally present at the nest site only when feeding chicks. Feedings may occur multiple times per day.

### Study area

**Figure 1:**
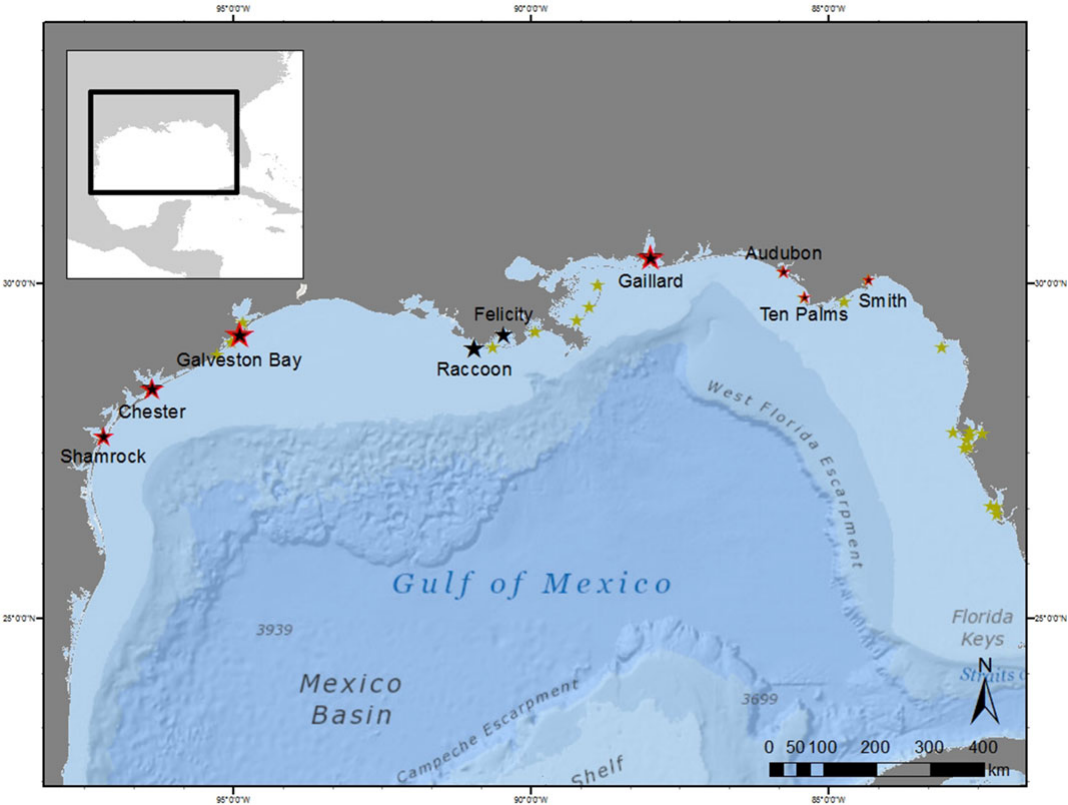
Location of brown pelican colonies sampled in the northern Gulf of Mexico. Marker sizes represent relative colony size (75–5000 nesting pairs). Nestling health samples were collected from all colonies, and nutrition and productivity data were also collected from colonies outlined in red. Locations of other brown pelican nesting colonies in the region are indicated in yellow.

We studied breeding brown pelicans between 2013 and 2015, throughout the northern Gulf of Mexico (Fig. 1). We selected colony sites to represent the full geographical range of pelican breeding areas in the region, with the exception of South Florida. In 2013, we collected physical measurements and feather samples from 3- to 4-week-old chicks at six colonies: two in the Florida panhandle, two in the Louisiana delta, and two along the central Texas coast. In 2014, we conducted chick sampling and monitored nest productivity at four colonies along the central and northern Texas coast. In 2015, we conducted chick sampling and monitored nest productivity at three colonies in the Florida panhandle and one in Alabama. Each colony site was sampled during a single breeding season.

### Nestling body condition

We randomly selected 3- to 4-week-old nestlings for sampling based on either hatch dates (where known) or plumage development (fully developed scapular contour feathers, remiges and rectrices in pin). If multiple nestlings were present, we sampled all siblings and determined hatch order based on culmen length (i.e. culmen length was assumed to be greater in first-hatched than second-hatched chicks, and in second-hatched than third-hatched chicks; Eggert and Jodice, 2008). Nestlings were readily captured by hand at or near nest sites. We collected physical measurements (culmen length, tarsus length, wing chord and mass), checked for the presence of ectoparasites, and counted all ticks found on the underside of the left wing. We also banded each chick on the right tarsus with a uniquely numbered stainless-steel US Geological Survey Bird Banding Lab leg band.

To calculate BCI, we conducted a principal components analysis on the three measures of skeletal size we collected: tarsus length, culmen length and wing chord (Benson *et al*., 2003). Using each individual's score on the first principal components axis (PC1) as an index of overall skeletal size, we calculated the best-fitting regression equation for the relationship between mass and principal component score. We chose a second-order polynomial to represent accurately the nestling growth process, which is initially linear but reaches a peak and descends slightly prior to fledging (Supplementary material, Fig. S1). Finally, we calculated BCI as the standardized residual of actual body mass from the value predicted by the regression equation.

### Feather corticosterone

At capture, we collected three or four scapular contour feathers from each nestling. Feathers were bagged and stored at room temperature until processing. We used random number generation to select 150 samples per year for CORT analysis, divided equally among study colonies. Following the recommendations of Lattin *et al*. (2011), we restricted the range of sample sizes analysed by excluding from analysis samples that were extremely small (<20 mg) and dividing samples larger than 160 mg into separate units for analysis. Mean total feather mass per sample was 87.8 (±41.6) mg, and mean total length per sample was 228.9 (±77.1) mm.

We closely followed the methods for feather CORT extraction and analysis originally described by Bortolotti *et al*. (2008). Briefly, we removed the calamus from each feather, weighed and measured feathers individually, and prepared the sample for analysis by snipping feathers into small (<0.5 mm) pieces with scissors and transferring the entire sample into a 16 ml test tube. Each sample received 7 ml of HPLC grade methanol (VWR) and was placed in a sonicating water bath at room temperature for 30 min, then transferred to a 50°C shaking waterbath overnight with caps added to tubes to limit evaporation. After centrifuging tubes, we used disposable glass Pasteur pipettes [Fisherbrand 5.75 inch (14.6 cm) or 9 inch (22.86 cm) and bulb, one use per pipette] to transfer methanol extracts to 13 ml tubes and conducted two additional washes of the feathers, each with 2.5 ml methanol and each for 2 h. Each cumulative methanol extraction sample, totaling 12 ml, was dried down under a nitrogen evaporator rack in a water bath at 40°C, reconstituted in 200 µl buffer, and put on a rack shaker for an hour before aliquoting. We conducted a radioimmunoassay (MP Biomedicals, LLC; ImmuniChem™ Double Antibody Corticosterone ^125^I RIA Kit) following the included directions, modified by using half-volumes and a 1:4 dilution for our samples (12.5 µl sample + 37.5 µl buffer). A parallelism test validated the use of the MP Bio kit with serial dilutions of samples parallel to the standard curve and a 1:4 dilution at 65% binding and near the middle of the curve. The intra-assay coefficient of variation using duplicate samples was 1.7–2.2%, and inter-assay variation of pooled feather samples was 11%. We assessed feather CORT in a total of 365 chicks (2013, *n* = 126; 2014, *n* = 144; and 2015, *n* = 95).

An important consideration in measuring CORT in feathers is whether to normalize CORT concentrations by sample length or sample mass (Bortolotti *et al*., 2008). Elevated CORT levels can result in lower feather quality (mass per unit length) that may obscure the relationship between environmental conditions and CORT deposition in feathers (Patterson *et al*., 2015). Given that feather quality was negatively correlated with feather CORT in our samples (*P* < 0.001, slope = −1.14 ± 0.15), we chose to normalize feather CORT using a measure of sample mass (in picograms per milligram) logarithmically transformed to meet assumptions of normality. To adjust for feather quality, we calculated the residual of the best-fitting regression line between logarithmically transformed CORT per milligram and feather mass per unit length, de-trended the data by subtracting the regression line, and used the adjusted values in all analyses. As feather quality is also positively correlated with nestling mass and nutritional intake (Patterson *et al*., 2015), normalizing by mass without adjusting for feather quality would exaggerate the strength of relationships between feather CORT, nutritional stress and fledging success.

### Nutritional stress

Nutritional stress in nestlings is the product of three component metrics: feeding rate (expressed as number of meals per chick per day), meal mass (in grams per meal) and energy density of prey (in kilojoules per gram). Following field methods used in previous studies (e.g. Jodice *et al*., 2006), we measured each of these metrics at the population level (breeding colony) and combined them to obtain an overall index of total daily energy delivery to nestlings (energy provisioning rate; EPR) for each study colony.

To measure feeding rates, we opportunistically selected groups of 15–20 nests at each colony visit and conducted 3 h observations, recording nest contents, arrival and departure times of adults, and feedings observed. Although we did not attempt to associate feeding rates with specific nests used for productivity and chick condition analysis, we selected nest groups in the same areas of the colony to ensure that we were sampling the same population. We considered a feeding to have occurred when a nestling inserted its head into the adult's gular pouch and emerged with its throat engorged (Sachs and Jodice, 2009). Pelican nestlings are fed multiple times per day during daylight hours, and we observed an average of 0.17 feedings per chick h^−1^ across all colonies, or ~2.5 feedings day^−1^ for each nestling, with colony-specific averages ranging from 1.5 to 3.2 feedings per chick day^−1^. We did not observe extensive self-feeding by nestlings, and thus considered only direct feedings from adults to nestlings. To measure meal mass, we collected 8–10 regurgitated meals from nestlings at each colony every 5–7 days, varying the timing and location of collection opportunistically. We obtained regurgitates by approaching nestlings and collecting meals that were regurgitated voluntarily. All collected samples were stored in plastic bags and frozen for later analysis.

In the laboratory, we thawed each sample in a warm-water bath, dried off surface water using paper towels, then weighed, measured and identified to species each individual fish. We classified each fish as whole (no visible damage), partial-whole (total length obtained, but some soft tissues missing) or partial (total length could not be obtained). For samples containing large numbers of fish (50–1000 items per sample; 26% of samples), we counted the total number of individuals of each species, weighed and measured a subsample of 10 individual fish per species, and obtained a total weight and overall classification (whole, partial-whole or partial) for each species group. For samples containing extremely large numbers of fish (>1000 items per sample; <1% of samples), we weighed and measured a subsample of 10 fish per species, weighed the overall sample, and used the average weight per fish to approximate the total number of fish in the sample. We did not analyse samples for which the digestive process was too advanced to identify fish to species (1% of samples). To estimate the mass of partial-whole and partial fish, we calculated the length–weight relationship as the best-fitting regression equation between the logarithm of total length and the logarithm of the mass of whole fish for each species by year (Supplementary material, Table S1). For partial-whole fish (i.e. degraded fish for which we were able to measure total length), we used the regression line to estimate the corrected mass of the whole fish from its length. For partial fish (i.e. degraded fish for which total length was not measurable), we used the mean total length of whole and partial-whole individuals collected from the same breeding colony on the same day to estimate a corrected mass from the regression equation.

We measured proximate composition and energy densities in whole samples (bait fish and undamaged chick regurgitates) of the most common prey fish species using extraction techniques as described by Anthony *et al*. (2000). Briefly, we dried fish to determine water content, extracted lipids from dried fish to determine lipid content, and ashed lean dry fish to determine protein content. The energy density for each prey item was then calculated as the sum of energy for lipid and protein. Species for which we were able to measure energy densities directly comprised 93% by biomass of all prey samples (Supplementary material, Table S2). For less-common species (7% of total biomass), we substituted either energy density values from other species within the same family (4%) or, if no comparable values were available in our data or in the literature, biomass-weighted averages of all other prey species (3%). We calculated the total energy content of sampled meals based on mean energetic values for each prey species multiplied by biomass, then averaged over the total meal mass to obtain a value in kilojoules per gram.

### Nest productivity and nestling survival

We visited nesting colonies close to the end of the incubation period and selected three or four groups of focal nests per colony, each group containing 20–30 nests. In colonies containing both elevated and ground nests, we selected closely spaced groups such that each contained nests of one type or the other to allow for comparison. On our initial visit, we recorded nest contents, assigned an identifying number to each nest, and photographed the nest group from marked observation points that could be accessed without disturbance to focal nests. On return visits, we identified nests using the numbered photograph and checked the contents of each nest from the observation point. Once nestlings reached 3–4 weeks of age, concurrent with measurements and feather sampling, we banded nestlings on the left tarsus with a permanent plastic band (Haggie Engraving: 2014, green; and 2015, blue) engraved with a three-digit white alpha code to aid in resighting.

Once nestlings began to disperse away from nest locations, we searched the surrounding areas of the colony with binoculars for banded chicks and recorded all bands observed. We continued observations until chicks reached at least 60 days of age. Beginning ~8 weeks after hatching, we also conducted regular searches of the colony for dead banded chicks and recovered all bands found. To determine nest productivity (fledglings per nest), nestlings that were observed alive at least 60 days after hatching and disappeared from the colony, but were not found dead, were presumed to have fledged successfully (Shields, 2014). We calculated plot- and colony-wide fledging success as the number of chicks fledged from observation nests, divided by the total number of nests observed.

To determine survival post-fledging, we relied on opportunistic resighting of banded chicks by colony monitors and birders along the coast of the Gulf of Mexico. We received band resightings and recoveries reported to the Bird Banding Lab, as well as directly to us through a dedicated Web portal. Sightings and recoveries were obtained throughout the US Gulf Coast and from Mexico until January 2016, representing a total of 88 unique individuals (15% of all colour-banded individuals). Post-fledging detection rates varied between colonies (6–20%), and we included a parameter for detection probability in survival models.

### Statistical analyses

We visually assessed frequency distributions of measured variables and, where necessary, used logarithmic transformations to meet assumptions of normality. To evaluate the relationship between CORT and BCI as predictors of individual survival to fledge, we conducted logistic regression with a binary outcome (fledged or died) on each metric and assessed the fit of the resulting models. To assess the relationships between CORT, BCI, nest-specific factors and individual survival, we ran independent generalized linear models, each with a binary outcome (fledged or died; and resighted alive or recovered dead) and logit link. We used CORT, BCI, nest elevation (ground or elevated), nesting colony, date, hatch order and number of siblings as fixed factors.

To calculate colony-wide survival rates, we used a joint live recapture–dead recovery model (Burnham, 1993), which accounted for differences in detection probability between colonies as well as differences in survival. We assessed survival rates at two time steps: survival to fledge (3 months after hatching) and post-dispersal survival (6 months after hatching). Dead individuals were recovered in the intervals between time steps, and individuals were considered to have survived to a new time step if they were resighted alive after that period ended. Given that resightings and recoveries took place across the entire range of the population, we fixed dispersal parameters (*F*) at 1 (i.e. 100% probability that banded individuals remained in the sampling area). We derived parameter estimates for survival (*S*), recovery (*r*) and resighting (*p*) during each time interval using Markov chain Monte Carlo estimators with a burn-in of 1000 samples, followed by 4000 tuning samples and 10 000 runs.

To compare the relative value of different metrics (CORT and BCI) for predicting aggregate nest productivity and survival rates, we used a generalized linear modelling framework (Gamma, log link), with fledging success as the response variable and average CORT, average BCI, and the interaction of CORT with BCI as predictor variables. We computed Akaike's Information Criterion for small samples (AICc) values to account for the small sample sizes that resulted from using colony as the sampling unit and used these values for model comparison. Models were considered to receive strong support if they resulted in a ΔAIC_c_ ≤ 2 and moderate support if they resulted in a ΔAIC_c_ of between 2 and 4 (Burnham and Anderson, 2004).

To assess nutritional stress by colony, we calculated the meal mass (in grams per meal), nestling provisioning rate (in meals per chick per hour) and energy density of meals (in kilojoules per gram) for each colony. These three components together form the energy provisioning rate (EPR, in grams per chick per hour; Jodice *et al*., 2006). To obtain a combined measure of EPR by colony, we modelled energy-days for each colony by randomly selecting (with replacement) 100 values for provisioning rate (in meals per day) from the set of measured values. The model then chose at random (with replacement) a mass and an energetic value for each meal, multiplied meal mass by energy density to obtain total energy content per meal, and summed total energy across all meals for each modelled day to obtain a set of energy provisioning rates (in kilojoules per day). We calculated the mean and standard deviation of EPR for each colony by averaging values obtained from 1000 runs of the model. We used generalized linear models (Gamma, log link) to assess the relationships of EPR and its component metrics to chick health parameters and nest productivity.

## Results

### Individual survival

**Figure 2:**
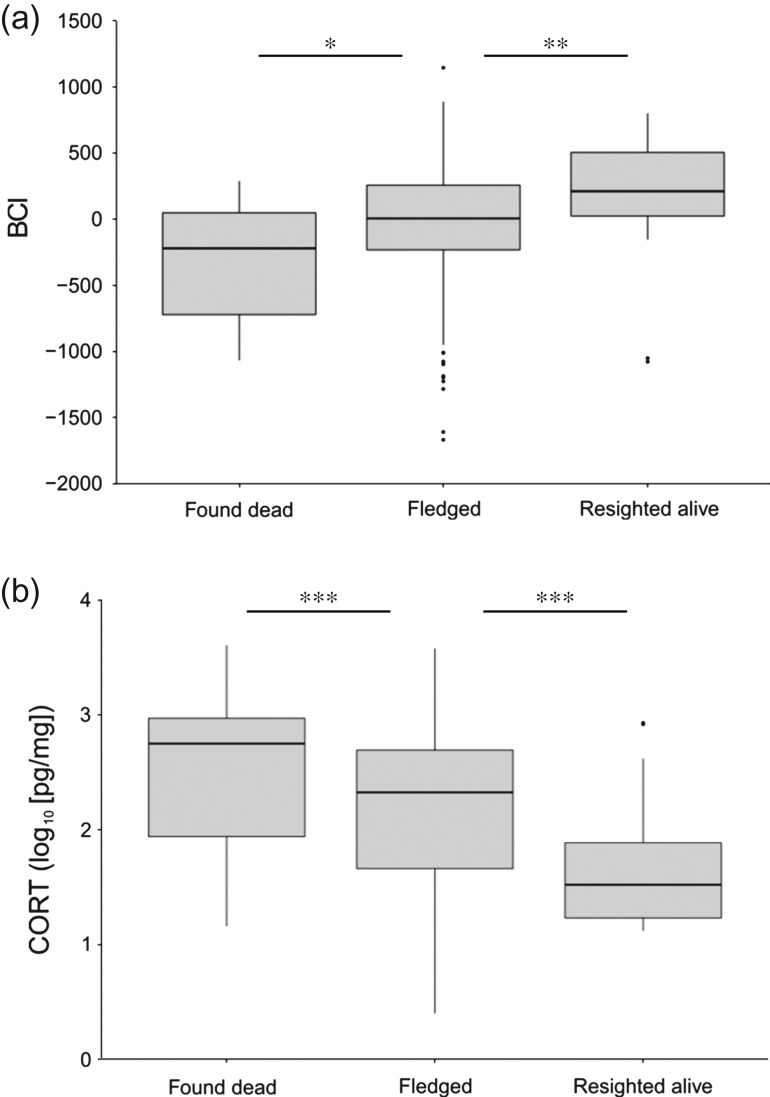
Distribution of individual measurements of body condition index (BCI; **a**) and feather corticosterone (CORT; **b**) at 3–4 weeks post-hatch for brown pelican nestlings later found dead after banding, presumed fledged and resighted alive after leaving the breeding colony in the northern Gulf of Mexico, 2013–15. Significant between-group differences are indicated by asterisks (*P < 0.05, **P < 0.01 and ***P < 0.001).

For individual nestlings, feather CORT concentrations were significantly negatively correlated to BCI (linear model: coefficient = −194 ± 31.6, *F*_1,364_ = 37.7, *P* < 0.001, *R*^2^ = 0.09). Chicks that died before fledging had lower BCI (*F*_1,239_ = 6.1, *P =* 0.01) and higher CORT deposited in feathers (*F*_1,239_ = 24.7, *P* < 0.001) at 3–4 weeks of age than chicks that were presumed fledged (i.e. survived until at least 60 days after hatching; Fig. 2). Of the other covariates we tested, only nest height (linear model, ground relative to elevated: coefficient = −2.79 ± 0.80, *z*_109_ = −3.76, *P* < 0.001) and body size (linear model: coefficient = 1.25 ± 0.43, *z*_109_ = 2.88, *P =* 0.004) were significantly correlated with individual fledging success. Nestlings from ground nests had significantly lower BCI (ground, μ_74_ = −97.2 ± 479; elevated, μ_117_ = 72.0 ± 363; *F*_1,191_ = 7.74, *P =* 0.006) and higher feather CORT (ground, μ_74_ = 2.08 ± 0.71; elevated, μ_117_ = 1.72 ± 0.64, *F*_1,191_ = 17.8, *P* < 0.001) than nestlings from elevated nests. We did not find a significant effect of colony, region, year, sampling date, hatch order, number of siblings, or their interactions on fledging probability (linear models: *P* > 0.10 for each).

Survival probabilities of individual nestlings >60 days post-hatch were positively related to BCI and negatively related to CORT (Fig. 2). Chicks found dead at the colony post-fledging had significantly lower BCI (ANOVA: *F*_1,40_ = 11.4, *P =* 0.002) and significantly higher CORT (ANOVA: *F*_1,40_ = 18.4, *P* < 0.001) at 3–4 weeks after hatching than did chicks that were resighted alive after fledging.

### Colony-specific nest productivity and chick survival

**Figure 3:**
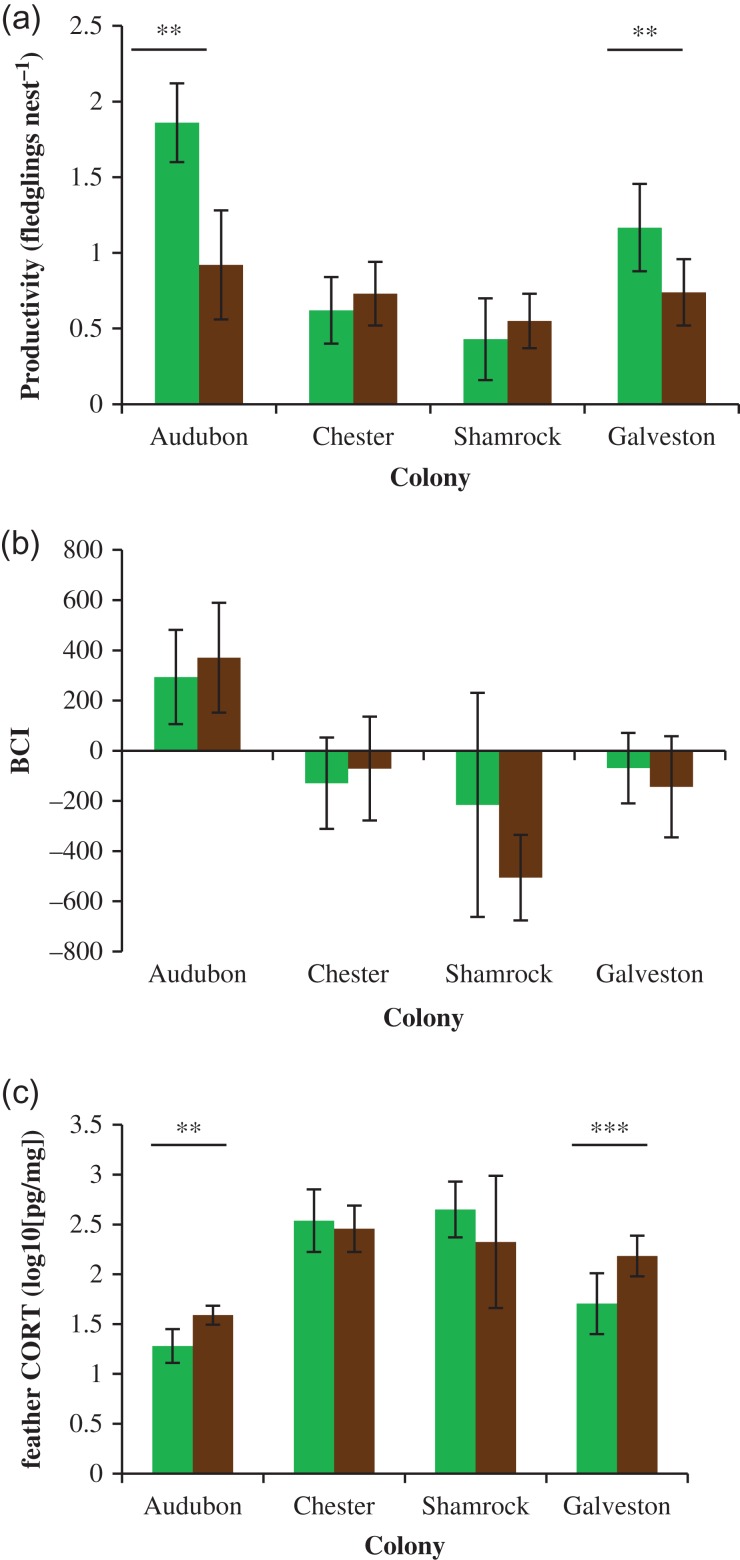
Mean values for brown pelican nest productivity (**a**), body condition index (BCI; **b**) and feather corticosterone (CORT; **c**) of nestlings in elevated (green) and ground (brown) nest plots in the northern Gulf of Mexico, 2014–15. Significant within-colony differences are indicated by asterisks (*P < 0.05, **P < 0.01 and ***P < 0.001); for all other differences, *P* > 0.05. Error bars represent 95% confidence intervals of the mean.

**Figure 4:**
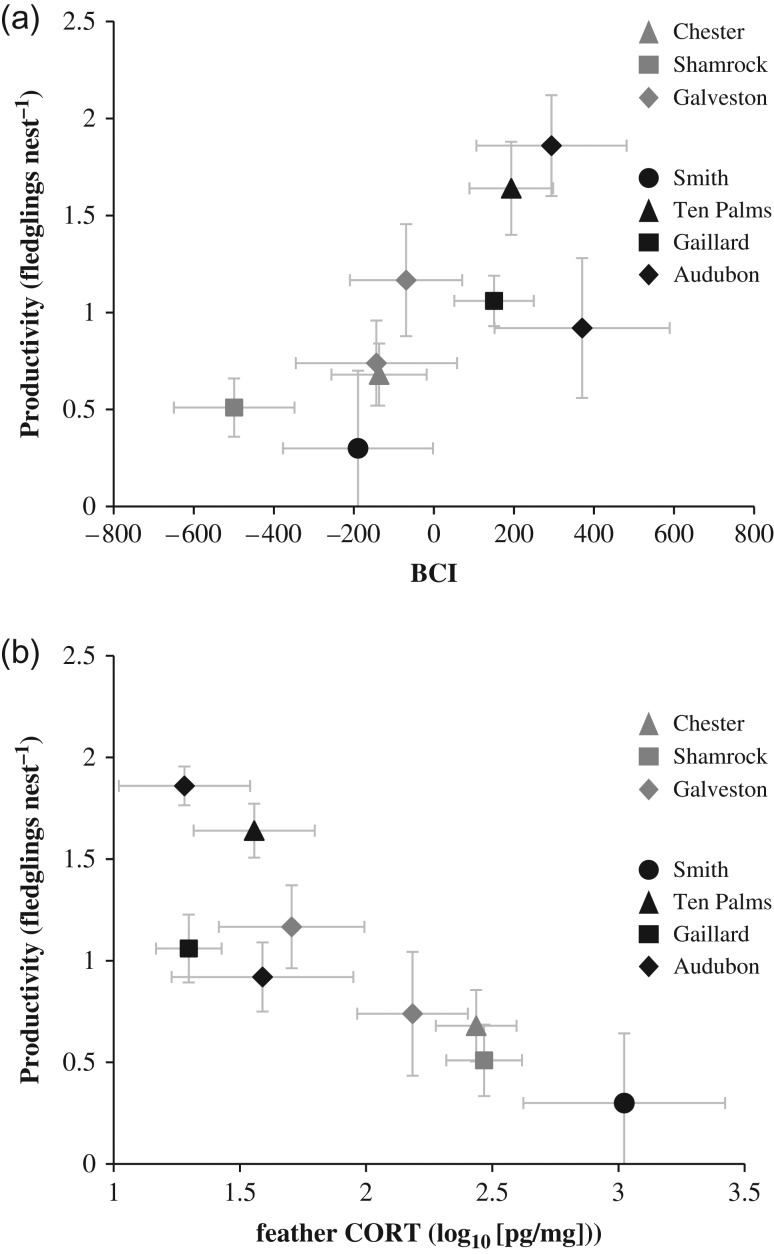
Correlation of mean brown pelican nest productivity to chick condition (BCI; **a**) and feather corticosterone (CORT; **b**) for colonies in the northern Gulf of Mexico, 2014–15. Points represent colony-wide averages except where different habitat types differed significantly in productivity, in which case mean values are separated by habitat type. Error bars represent 95% confidence intervals.

Within breeding colonies, CORT levels were correlated with nest productivity at individual observation plots. Nest productivity (Fig. 3a) and nestling feather CORT (Fig. 3c), but not nestling BCI (Fig. 3b), differed significantly between ground and elevated subplots at two of the four colonies with both ground and elevated nests. Of the remaining three colonies, two contained only shrub nests and the third contained too few ground nests to assess differences in productivity relative to shrub nests. Overall, colony-wide productivity rates were positively correlated with average BCI (Fig. 4a) and negatively correlated with average CORT (Fig. 4b) of sampled chicks. The strongest model predicting colony-specific nest productivity as a function of chick health parameters, which was also the only model supported by comparison of AIC_c_ values, contained CORT alone (Table 1). The top model explained 84% of the observed deviance (null = 1.91; residual = 0.31).

**Table 1:** Candidate models for brown pelican nest productivity and nestling survival in the northern Gulf of Mexico as a function of colony-average body condition index (BCI) and feather corticosterone (CORT) of 3- to 4-week-old chicks, ranked in order of increasing Akaike information criterion (AIC_c_) values with model weights (*w*_i_), cumulative weights (Σ*w*) and relative likelihoods (*L*_i_)

	Terms	AIC_c_	Δ_i_ (AIC_c_)	*w*_i_ (AIC_c_)	Σ*w*	*L*_i_ (AIC_c_)
Productivity						
	**CORT**	**4.17**	**0**	**0.94**	**0.94**	**1.00**
	BCI	11.14	6.97	0.03	0.97	0.03
	BCI + CORT	11.40	7.22	0.02	0.99	0.02
	Null model	13.06	8.88	0.01	1.00	0.01
Post-banding survival						
	**CORT**	**−17.66**	**0**	**0.96**	**0.96**	**1.00**
	BCI + CORT	**−**10.70	6.87	0.03	0.99	0.03
	BCI	**−**6.34	11.32	<0.01	1.00	<0.01
	Null model	**−**5.40	12.27	<0.01	1.00	<0.01
Post-dispersal survival						
	**CORT**	**−19.80**	**0**	**0.55**	**0.55**	**1.00**
	**Null model**	**−18.74**	**1.06**	**0.32**	**0.87**	**0.59**
	BCI	**−**16.53	3.27	0.11	0.98	0.19
	BCI + CORT	**−**13.00	6.81	0.02	1.00	0.03

Models in bold were considered strongly supported.

**Figure 5:**
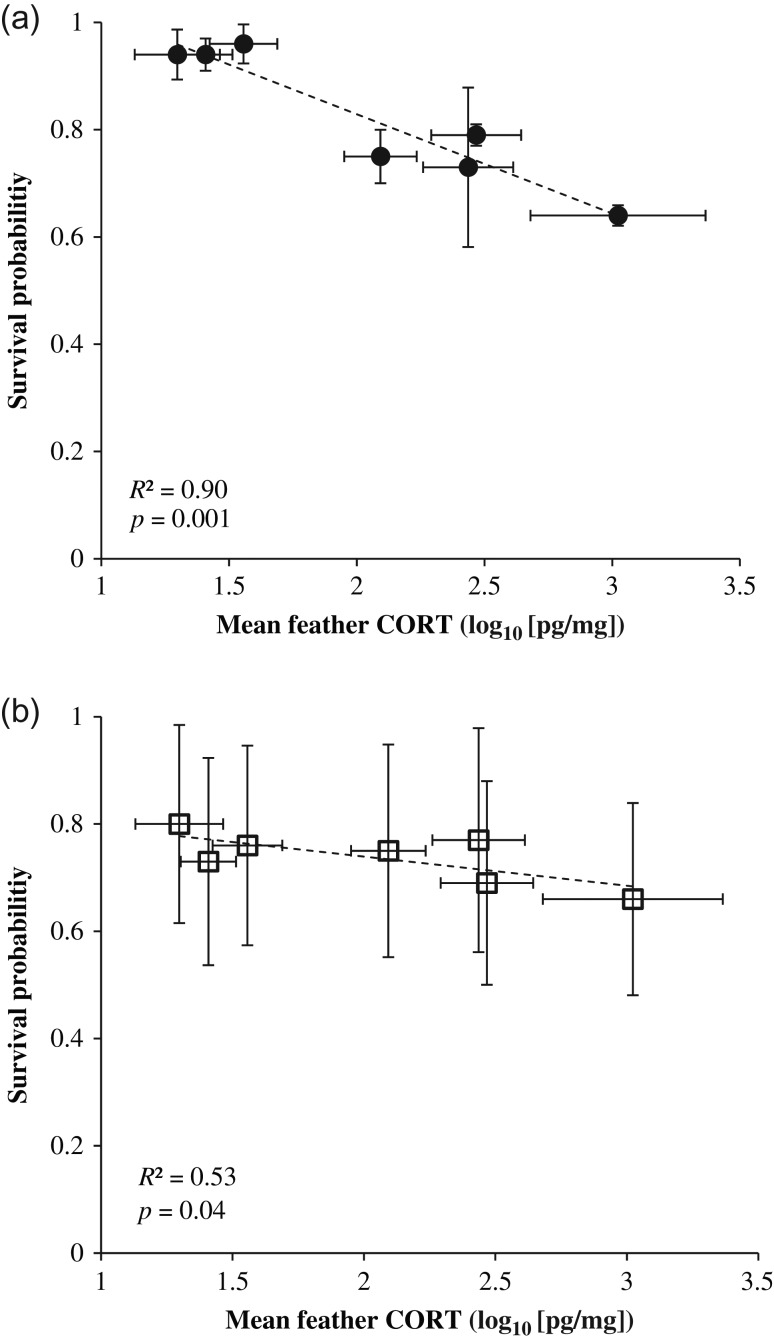
Relationship of colony-wide mean brown pelican nestling feather corticosterone (CORT) to probability of survival to fledge (**a**) and post-dispersal survival (**b**) in the northern Gulf of Mexico, 2014–15. Error bars represent 95% confidence intervals.

Modelled chick survival to fledge (3 months after hatch) at individual colony sites was negatively correlated with average CORT (Fig. 5a). The strongest model predicting chick survival to fledge as a function of chick health parameters, which was also the only model supported by comparison of AIC_c_ values, contained CORT alone (Table 1). The top model explained 91% of the observed deviance (null = 0.144; residual = 0.013). The relationship between BCI and survival to fledge showed a non-significant positive trend, and BCI was not supported as a predictor of average colony-wide survival rates (Table 1).

Average CORT values at individual colony sites were negatively correlated with modelled chick survival post-dispersal (to 6 months after hatching; Fig. 5b). Both the CORT-only model and the null model were supported as predictors of post-dispersal survival, although the former was 1.7 times as likely as the latter to be the best model (Table 1). The top model explained 48% of the observed deviance (null = 0.026; residual = 0.012). Body condition index was not included in any supported models of post-dispersal survival.

### Nutritional stress

**Figure 6:**
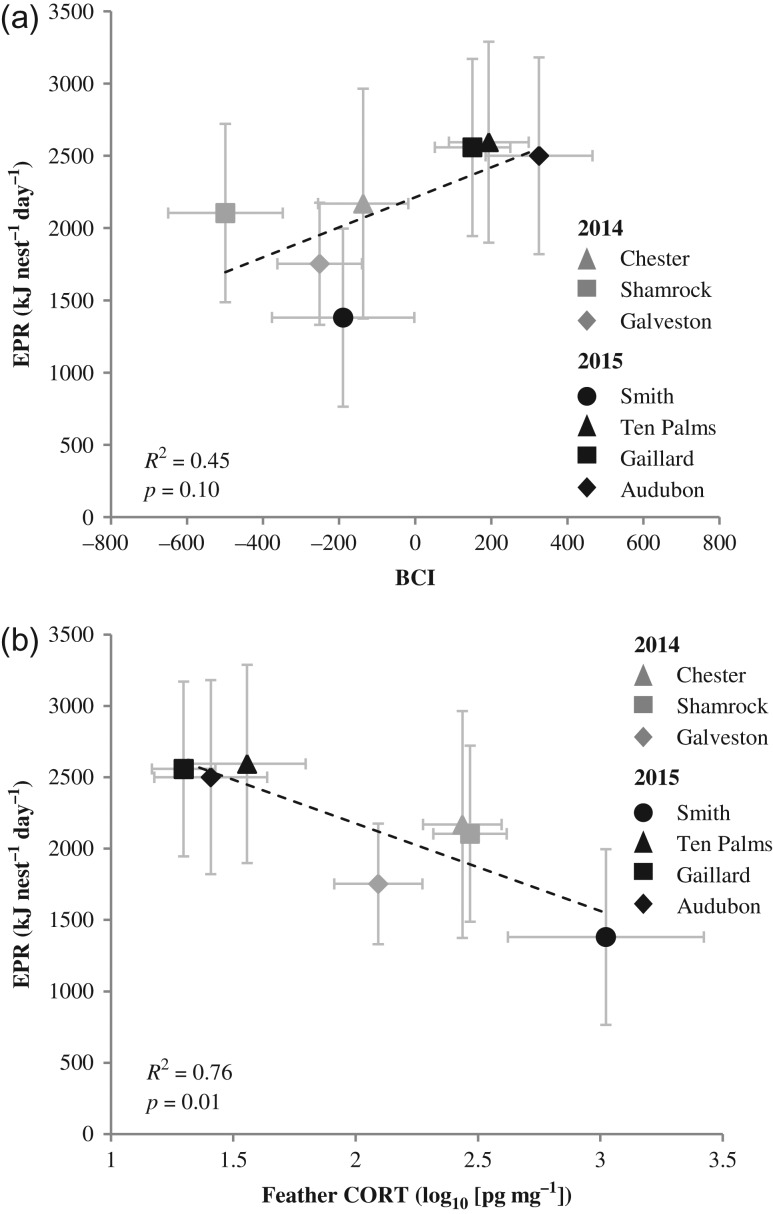
Relationship of brown pelican energy provisioning rate (EPR) to chick health parameters body condition index (BCI; **a**) and feather corticosterone (CORT; **b**) by colony in the northern Gulf of Mexico, 2014–15. Points represent colony-wide mean values, and error bars represent 95% confidence intervals.

Energy provisioning rate showed a non-significant positive trend in relationship to BCI (linear model, coefficient = 1.04 ± 0.52, *t*_5_ = 2.02, *P =* 0.10; Fig. 6a) and a significant negative relationship to feather CORT (linear model, coefficient = −613 ± 155, *t*_5_ = 3.97, *P =* 0.01; Fig. 6b). The two biomass components of EPR, feeding frequency (meals per chick day^−1^, μ = 2.51, *n* = 142) and meal mass (grams per meal, μ = 157.6, *n* = 583) had similarly high levels of overall variation [coefficient of variation (CV) frequency = 0.64; CV mass = 0.76], whereas energy density of meals (kilojoules per gram, μ = 4.34, *n* = 583) was less variable (CV = 0.10). Energy provisioning rate explained 76% of observed variance in colony-wide average feather CORT and 45% of observed variance in colony-wide average BCI (Fig. 6). Of the separate components of EPR (Table 2), meal delivery rate explained the largest portion of variance in each of the two chick health metrics (CORT, 30.5%; and BCI, 33.0%), followed by meal mass (CORT, 22.1%; and BCI, 3.7%) and energy density (CORT, 3.2%; and BCI, 0.1%). Energy provisioning rate was positively correlated with nest productivity (coefficient = 739 ± 258, *t*_5_ = 2.85, *P* < 0.04, *R*^2^ = 0.62) and nestling survival to fledge (coefficient = 3365 ± 580, *t*_4_ = 5.80, *P =* 0.002, *R*^2^ = 0.87), and the relationship between EPR and post-fledging survival rates showed a positive but non-significant trend (coefficient = 6482 ± 3042, *t*_4_ = 2.13, *P =* 0.09).

**Table 2:** Mean values (±SD) for brown pelican nest productivity, chick health metrics and energy provisioning metrics by colony in the northern Gulf of Mexico 2014–15

	Colony	Productivity	Body condition index	Corticosterone	Meals day^−1^	Grams per meal	Energy g^−1^	Energy provisioning rate
2014	Shamrock	0.51 ± 0.66	**−**499 ± 446	2.47 ± 0.52	1.63 ± 0.94	205 ± 138	4.66 ± 0.50	2094 ± 1374
2014	Chester	0.68 ± 0.79	**−**136 ± 372	2.44 ± 0.54	2.28 ± 2.25	174 ± 136	4.53 ± 0.61	2164 ± 2062
2014	Galveston	0.94 ± 0.86	**−**251 ± 472	2.09 ± 0.60	3.15 ± 1.50	124 ± 91	3.99 ± 0.63	1750 ± 1031
2015	Smith	0.30 ± 0.64	**−**189 ± 209	3.02 ± 0.38	2.49 ± 1.22	106 ± 78	4.35 ± 0.39	1383 ± 810
2015	Ten Palms	1.64 ± 0.95	193 ± 291	1.56 ± 0.37	2.95 ± 1.68	168 ± 105	4.59 ± 0.35	2584 ± 1539
2015	Audubon	1.42 ± 0.85	325 ± 379	1.41 ± 0.28	2.49 ± 1.05	191 ± 170	4.33 ± 0.38	2500 ± 1562
2015	Gaillard	1.06 ± 0.85	150 ± 272	1.30 ± 0.46	2.61 ± 1.24	175 ± 102	4.69 ± 0.36	2561 ± 1300

## Discussion

We found that corticosterone in nestling feathers, which represents an integrated measure of developmental stress during feather growth, was highly correlated with traditional measures of reproductive success (fledglings per nest) and nestling health (BCI) at individual, subcolony and colony-wide scales. Moreover, our results indicate that measuring feather corticosterone in young, altricial chicks can explain differences in chick health, fledging success and post-fledging survival that are not captured by body condition alone.

Our first objective was to assess the relationship between feather CORT and a more traditional measure of nestling health, BCI (Benson *et al*., 2003), as predictors of nestling survival. In accordance with recent work on other avian taxa, we found that nestling feather CORT was negatively correlated with both body condition (Fairhurst *et al*., 2013; López-Jiménez *et al*., 2016) and fledging probability (Fairhurst *et al*., 2013; Lodjak *et al*., 2015) at the individual level. Although both feather CORT and BCI were significantly correlated with chick survival to fledge, feather CORT predicted the fate of individual nestlings slightly better compared with BCI. At the colony level, models containing only feather CORT were favoured over models containing BCI with and without feather CORT as predictors of nest productivity, survival to fledge and post-dispersal survival. Additionally, feather CORT predicted within-colony differences in fledging success by habitat type that were not apparent in comparisons of BCI. The enhanced explanatory power of CORT compared with BCI may be attributable to both the longer time frame over which CORT integrates physiological condition and the sensitivity that BCI has to short-term variation in nutritional stress. For example, at the Shamrock Island colony the average mass of chicks was 2660 g and average meal mass 181 g, or about 7% of body weight. This relatively high ratio of meal mass to body mass, combined with the daily variation we observed in the mass of meals, makes BCI highly sensitive to feeding frequency and time since feeding. Meal delivery rates and the size of meals in relationship to chick mass can vary by more than one order of magnitude both among and within avian species (Ricklefs *et al*., 1985; Anderson and Ricklefs, 1992), so the use of BCI as a measure of nestling condition requires consideration of how these short-term factors may influence its utility in describing long-term patterns of chick condition. Feather CORT integrates a longer time series of conditions (Bortolotti *et al*., 2008) and thus may be less susceptible than BCI to short-term variation. The fact that we measured feather CORT early in development (~20–30 days into a 60–90 day nesting period) and found a strong relationship to fledging probability further indicates that feather CORT levels during early development can accurately predict survival through the breeding season.

We also assessed the relationship between feather CORT and variation in local (site- and nest-specific) conditions. Although nestling feather CORT is strongly correlated with environmental conditions during development (e.g. Harms *et al*., 2010; Lodjak *et al*., 2015; Will *et al*., 2015), site- and nest-specific factors can still confound the environment–stress relationship (Fairhurst *et al*., 2012; Lodjack *et al*., 2015). We did not find a significant influence of either hatch order or number of siblings on feather CORT. A previous study of plasma CORT in brown pelican nestlings (Eggert *et al*., 2010) also found no effect of brood size or hatch order on stress levels; however, sibling dynamics have been found to affect feather CORT levels in nestling raptors (Yosef *et al*., 2013; López-Jiménez *et al*., 2016). We did find an influence of microhabitat characteristics (elevated vs. ground nest location) on feather CORT. Nestlings at elevated nests might benefit from improved passive thermoregulation, reduced energy expended in movement, and reduced aggressive interactions with neighbouring adults and nestlings that subsequently act to maintain lower levels of feather CORT. Our study concurs with data on brown pelican nest productivity in Louisiana (Walter *et al*., 2013), suggesting that nestlings from elevated nests tend to survive longer than nestlings from ground nests, contributing to increased nest productivity at elevated sites. If elevated nest sites offer improved fledging success, positive reinforcement may occur at these sites if experienced or dominant breeders preferentially select and defend elevated nesting sites.

Finally, we tested the relationship between nestling health metrics, nutritional stress (EPR) and breeding success. Pelicans in our study area rarely experience nest predation, human disturbance or extreme weather events during breeding; hence, few factors are likely to confound the relationship between developmental stress and chick mortality. Our results indicated that both nestling feather CORT and nestling BCI were highly correlated with EPR, and that EPR explained 87% of the variation in chick survival between the colonies we studied. Of the components of EPR, meal delivery rate explained a larger portion of the variance in survival metrics and nestling health than did meal mass or energy density of prey. Meal mass also explained a high proportion of variance in nestling feather CORT, although not BCI or survival, whereas energy density had no significant linear relationships with nestling health or survival metrics. The low correlation between nestling health and energy density in this system is in contrast to previous studies of seabirds (reviewed by Österblom *et al*., 2008) that have suggested prey quality as a key driver of nestling survival. We posit that the weaker relationship we observed may be attributable in part to a narrower range of energy content of prey in the Gulf of Mexico, particularly a lack of prey with the high levels of energy density and lipid content that occur in high-latitude systems (Stickney and Torres, 1989; Anthony *et al*., 2000). Once nestlings fledged, EPR at the natal colony was no longer a strong predictor of survival probability, indicating that differences in the quantity of food during development are not a dominant driver of survival after dispersal. However, both feather CORT and BCI were correlated with post-fledging survival, which suggests that nutritional stress during development may continue to influence the probability that individuals will survive to recruit back into the breeding population once they have fledged. The demographic effects of negative feedbacks between developmental stress and recruitment have been documented in other seabird species (e.g. Kitaysky *et al*., 2010). Linking these parameters is a necessary step toward understanding the long-term demographic consequences of perturbations in the developmental environment.

Given its ease of implementation and strong relationship to nestling survival, feather CORT is uniquely suited to detecting the effects of sublethal stress on reproductive success and can be collected rapidly in response to unexpected environmental perturbations. Although measuring feather CORT requires more post-collection laboratory analysis than traditional reproductive success and chick health metrics, its advantages include minimal disturbance at breeding colonies, ease of collection and storage, and the ability to sample multiple colonies in a short time. However, interspecific differences in the stress response may make this technique more suitable for some seabird species than others (e.g. Kitaysky *et al*., 2005). The existence of a detectable relationship between environmental covariates and nestling stress is a crucial prerequisite for using feather CORT as an indicator of environmental conditions. Moreover, to draw inferences at broad spatial scales (e.g. between colonies or regions), sampling regimes would need to account for the influence of varying habitat characteristics. Several recent studies of feather CORT, particularly Fairhurst *et al*. (2014), Lodjak *et al*. (2015) and López-Jiménez *et al*. (2016), have described the context dependence of the stress–environment relationship and its sensitivity to local-scale habitat quality and climatic variation. Our results indicate that, although sibling dynamics do not confound variation in feather CORT in this species, nest height can affect both physiology and survival. These differences highlight the importance of understanding how different site- and individual-specific factors contribute to underlying variation in measured parameters, and how these factors could interact cumulatively or multiplicatively with environmental conditions to mask or exaggerate the effects of perturbations on reproduction.

We found both inter- and intraregional variation in colony-specific nestling health and reproductive success in baseline conditions across the northern Gulf of Mexico. The foraging environment experienced by breeding seabirds depends on a variety of biotic and abiotic factors that can change across a species’ range as well as between and within breeding seasons. Distinguishing the effects of environmental perturbations requires that the effects of short-term changes to foraging conditions be distinguished from the background noise of pre-existing variation. End points that can be measured consistently across space and time provide a unifying approach for long-term monitoring efforts that can compare baseline measures with post-disturbance conditions. Our study provides evidence that feather CORT can be used to detect differences in underlying nutritional quality and predict reproductive parameters in a free-living seabird population, in which nestlings elevate stress hormone levels in response to nutritional constraints, making it an appropriate basis for long-term monitoring of population-wide reproductive health and, ultimately, detection of the indirect demographic effects of environmental change.

## References

[cow060C1] Almasi B, Roulin A, Jenni-Eiermann S, Breuner CW, Jenni L (2009) Regulation of free corticosterone and CBG capacity under different environmental conditions in altricial nestlings. Gen Comp Endocrinol 164: 117–124. 19467233 10.1016/j.ygcen.2009.05.011

[cow060C2] Anderson DJ, Ricklefs RE (1992) Brood size and food provisioning in masked and blue-footed boobies (*Sula* spp.). Ecology 73: 1363–1374.

[cow060C3] Angelier F, Schaffer S, Weimerskirch H, Trouve C, Chastel O (2007) Corticosterone and foraging behavior in a pelagic seabird. Physiol Biochem Zool 80: 283–292. 17390284 10.1086/512585

[cow060C4] Anthony JA, Roby DD, Turco KR (2000) Lipid content and energy density of forage fishes from the northern Gulf of Alaska. J Exp Mar Biol Ecol 248: 53–78. 10764884 10.1016/s0022-0981(00)00159-3

[cow060C5] Balseiro A, Espi A, Marquez I, Perez V, Ferreras MC, Marín JG, Prieto JM (2005) Pathological features in marine birds affected by the Prestige's oil spill in the north of Spain. J Wildlife Dis 41: 371–378. 10.7589/0090-3558-41.2.371 16107672

[cow060C6] Benson J, Suryan RM, Piatt JF (2003) Assessing chick growth from a single visit to a seabird colony. Mar Ornithol 31: 181–184.

[cow060C7] Blas J, Baos R, Borlotti GR, Marchant T, Hiraldo F (2005) A multi-tier approach to identifying environmental stress in altricial nestling birds. Funct Ecol 19: 315– 322.

[cow060C8] Bonier F, Martin PR, Sheldon KS, Jensen JP, Foltz SL, Wingfield JC (2007) Sex-specific consequences of life in the city. Behav Ecol 18: 121– 129.

[cow060C9] Bonier F, Moore IT, Martin PR, Robinson RJ (2009) The relationship between ﬁtness and baseline glucocorticoids in a passerine bird. Gen Comp Endocrinol 163: 208– 213. 19135999 10.1016/j.ygcen.2008.12.013

[cow060C10] Bortolotti GR, Marchant TA, Blas J, German T (2008) Corticosterone in feathers is a long-term, integrated measure of avian stress physiology. Funct Ecol 22: 494– 500.

[cow060C11] Bortolotti GR, Marchant T, Blas J, Cabezas S (2009) Tracking stress: localisation, deposition and stability of corticosterone in feathers. J Exp Biol 212: 1477– 1482. 19411541 10.1242/jeb.022152

[cow060C12] Burger AE (1993) Estimating the mortality of seabirds following oil spills: effects of spill volume. Mar Pollut Bull 26: 140– 143.

[cow060C13] Burnham KP (1993) A theory for combined analysis of ring recovery and recapture data In Lebreton JD, North PM, eds, Marked Individuals in the Study of Bird Populations. Birkhauser-Verlag, Basel, pp 199–213.

[cow060C14] Burnham KP, Anderson DR (2004) Multimodel inference: understanding AIC and BIC in model selection.Sociol Method Res 33: 261–304.

[cow060C15] Butler MW, Leppert LL, Duffy AM (2010) Effects of small increases in corticosterone levels on morphology, immune function, and feather development. Physiol Biochem Zool 83: 78– 86. 19929638 10.1086/648483

[cow060C16] Butler RG, Harfenist A, Leighton FA, Peakall DB (1988) Impact of sublethal oil and emulsion exposure on the reproductive success of Leach's storm-petrels: short and long-term effects. J Appl Ecol 25: 125–143.

[cow060C17] Cheng S, Kong J, Song H (2009) Wenchuan 512 earthquake and giant panda habitat in Wolong, China: a review of strong earthquake effects. Front For China 4: 388–393.

[cow060C18] Eggert LMF, Jodice PGR (2008) Growth of Brown Pelican nestlings exposed to sublethal levels of soft tick infestation. Condor 110: 134–142.

[cow060C19] Eggert LMF, Jodice PGR, O'Reilly KM (2010) Stress response of brown pelican nestlings to ectoparasite infestation. Gen Comp Endocrinol 166: 33–38. 19716827 10.1016/j.ygcen.2009.08.009

[cow060C20] Eppley ZA (1992) Assessing indirect effects of oil in the presence of natural variation: the problem of reproductive failure in South Polar skuas during the Bahia Paraiso oil spill. Mar Pollut Bull 25: 307–312.

[cow060C21] Fairhurst GD, Navarro J, González-Solís J, Marchant TA, Bortolotti GR (2012) Feather corticosterone of a nestling seabird reveals consequences of sex-specific parental investment. Proc Biol Sci 279: 177– 184. 21632628 10.1098/rspb.2011.0884 PMC3223659

[cow060C22] Fairhurst GD, Marchant TA, Soos C, Machin KL, Clark RG (2013) Experimental relationships between levels of corticosterone in plasma and feathers in a free-living bird. J Exp Biol 216: 4071– 4081. 23913947 10.1242/jeb.091280

[cow060C23] Fairhurst GD, Dawson RD, van Oort H, Bortolotti GR (2014) Synchronizing feather-based measures of corticosterone and carotenoid-dependent signals: what relationships do we expect. Oecologia 174: 689–698. 24233689 10.1007/s00442-013-2830-5

[cow060C24] Gannon MR, Willig MR (1994) The effects of Hurricane Hugo on bats of the Luquillo Experimental forest of Puerto Rico. Biotropica 26: 320–331.

[cow060C25] Haney JC, Geiger HJ, Short JW (2014) Bird mortality from the Deepwater Horizon oil spill. II. Carcass sampling and exposure probability in the coastal Gulf of Mexico. Mar Ecol Prog Ser 513: 239– 252.

[cow060C26] Harms NJ, Fairhurst GD, Bortolotti GR, Smits JE (2010) Variation in immune function, body condition, and feather corticosterone in nestling tree swallows (*Tachycineta bicolor*) on reclaimed wetlands in the Athabasca oil sands, Alberta, Canada. Environ Pollut 158: 841– 848. 19850385 10.1016/j.envpol.2009.09.025

[cow060C27] Jakob EM, Marshall SD, Uetz GW (1996) Estimating fitness: a comparison of body-condition indices. Oikos 77: 61– 67.

[cow060C28] Jodice PGR, Roby DD, Turco K, Suryan R, Irons DB, Roseneau D, Kettle A, Piatt J, Shultz M, Anthony JA (2006) Assessing the nutritional stress hypothesis: the relative influence of diet quantity and quality on seabird productivity. Mar Ecol Prog Ser 325: 267– 279.

[cow060C29] Kitaysky AS, Kitaiskaia EV, Wingfield JC, Piatt JF (2001) Diteary restriction causes chronic elevation of corticosterone and enhances stress response in red-legged kittiwake chicks. J Comp Physiol B 171: 701– 709. 11765979 10.1007/s003600100230

[cow060C30] Kitaysky AS, Kitaiskaia EV, Piatt JF, Wingfield JC (2003) Beneﬁts and costs of increased levels of corticosterone in seabird chicks. Horm Behav 43: 140– 149. 12614644 10.1016/s0018-506x(02)00030-2

[cow060C31] Kitaysky AS, Romano MD, Piatt JF, Wingfield JC, Kikuchi M (2005) The adrenocortical response of tufted puffin chicks to nutritional deficits. Horm Behav 47: 606– 619. 15811363 10.1016/j.yhbeh.2005.01.005

[cow060C32] Kitaysky AS, Piatt JF, Hatch SA, Kitaiskaia EV, Benowitz-Fredericks ZM, Shultz MT, Wingfield JC (2010) Food availability and population processes: severity of nutritional stress during reproduction predicts survival of long-lived seabirds. Funct Ecol 24: 625–637.

[cow060C33] Krebs CT, Burns KA (1977) Long-term effects of an oil spill on populations of the salt-marsh crab *Uca pugnax*. Science 197: 484–487. 17783248 10.1126/science.197.4302.484

[cow060C34] Labocha MK, Hayes JP (2012). Morphometric indices of body condition in birds: a review. J Ornithol 153: 1– 22.

[cow060C35] Lattin CR, Reed JM, des Rochers DW, Romero LM (2011) Elevated corticosterone in feathers correlates with corticosterone-induced decreased feather quality: a validation study. J Avian Biol 42: 1–6.

[cow060C36] Lodjak J, Mägi M, Rooni U, Tilgar V (2015) Context-dependent effects of feather corticosterone on growth rate and fledging success of wild passerine nestlings in heterogeneous habitat. Oecologia 179: 937– 946. 26025576 10.1007/s00442-015-3357-8

[cow060C37] López‐Jiménez L, Blas J, Tanferna A, Cabezas S, Marchant T, Hiraldo F, Sergio F (2016) Ambient temperature, body condition and sibling rivalry explain feather corticosterone levels in developing black kites. Funct Ecol 30: 605–613.

[cow060C38] Love OP, Shutt LJ, Silfies JS, Bird DM (2003) Repeated restraint and sampling results in reduced corticosterone levels in developing and adult captive American Kestrels (*Falco sparverius*). Physiol Biochem Zool 76: 753–761. 14671722 10.1086/376431

[cow060C39] McEwen BS, Biron CA, Brunson KW, Bulloch K, Chambers WH, Dhabhar FS, Goldfarb RH, Kitson RP, Miller AH, Spencer RL et al (1997) The role of adrenocorticoids as modulators of immune function in health and disease: neural, endocrine and immune interactions. Brain Res Rev 23: 79–133. 9063588 10.1016/s0165-0173(96)00012-4

[cow060C40] Marra PP, Holberton RL (1998) Corticosterone levels as indicators of habitat quality: effects of habitat segregation in a migratory bird during the non-breeding season. Oecologia 116: 284– 292. 10.1007/s004420050590 28308538

[cow060C41] Møller AP, Mousseau TA (2011) Conservation consequences of Chernobyl and other nuclear accidents. Biol Conserv 144: 2787–2798.

[cow060C42] Monaghan P, Heidinger BJ, D'Alba L, Evans NP, Spencer KA (2012) For better or worse: reduced adult lifespan following early-life stress is transmitted to breeding partners. Proc Biol Sci 279: 709–714. 21849320 10.1098/rspb.2011.1291 PMC3248736

[cow060C43] Müller C, Jenni-Eiermann S, Jenni L (2009) Effects of a short period of elevated circulating corticosterone on postnatal growth in free-living Eurasian kestrels *Falco tinnunculus*. J Exp Biol 212: 1405–1412. 19376961 10.1242/jeb.024455

[cow060C44] Österblom H, Olsson O, Blenckner T, Furness RW (2008) Junk‐food in marine ecosystems. Oikos 117: 967– 977.

[cow060C45] Patterson AG, Kitaysky AS, Lyons DE, Roby DD (2015) Nutritional stress affects corticosterone deposition in feathers of Caspian tern chicks. J Avian Biol 46: 18– 24.

[cow060C46] Peterson CH (2001) The “Exxon Valdez” oil spill in Alaska: Acute, indirect, and chronic effects on the ecosystem. Adv Mar Biol 39: 1–103.

[cow060C47] Peterson CH, Rice SD, Short JW, Esler D, Bodkin JL, Ballachey BE, Irons DB (2003) Long-term ecosystem response to the Exxon Valdez oil spill. Science 302: 2082– 2086. 14684812 10.1126/science.1084282

[cow060C48] Piatt JF, Lensink CJ, Butler W, Kendziorek M, Nysewander DR (1990) Immediate impact of the Exxon Valdez oil spill on marine birds. Auk 107: 387– 397.

[cow060C49] Ricklefs RE, Day CH, Huntington CE, Williams JB (1985) Variability in feeding rate and meal size of Leach's storm-petrel at Kent Island, New Brunswick. J Anim Ecol 54: 883– 898.

[cow060C50] Romero LM, Fairhurst GD (2016) Measuring corticosterone in feathers: strengths, limitations, and suggestions for the future.Comp Biochem Physiol A Mol Integr Physiol. 202: 112–122. 27155053 10.1016/j.cbpa.2016.05.002

[cow060C51] Romero LM, Reed JM (2005) Collecting baseline corticosterone samples in the field: is under 3 min good enough. Comp Biochem Physiol A Mol Integr Physiol 140: 73–79. 15664315 10.1016/j.cbpb.2004.11.004

[cow060C52] Romero LM, Wikelski M (2001) Corticosterone levels predict survival probabilities of Galápagos marine iguanas during El Niño events. Proc Natl Acad Sci USA 98: 7366– 7370. 11416210 10.1073/pnas.131091498 PMC34674

[cow060C53] Sachs EB, Jodice PGR (2009) Behavior of parent and nestling Brown Pelicans during early brood-rearing. Waterbirds 32: 276– 281.

[cow060C54] Sapolsky RM, Romero LM, Munck AU (2000) How do glucocorticoids influence stress responses? Integrating permissive, suppressive, stimulatory, and preparative actions. Endocr Rev 21: 55– 89. 10696570 10.1210/edrv.21.1.0389

[cow060C55] Shields M (2014) Brown pelican (*Pelecanus occidentalis*) In Poole A, ed, The Birds of North America Online. Cornell Laboratory of Ornithology, Ithaca http://bna.birds.cornell.edu/bna/species/609.

[cow060C56] Smits JE, Fernie KJ (2013) Avian wildlife as sentinels of ecosystem health. Comp Immunol Microb 36: 333– 342. 10.1016/j.cimid.2012.11.007 23260372

[cow060C57] Spencer KA, Evans NP, Monaghan P (2009) Postnatal stress in birds: a novel model of glucocorticoid programming of the hypothalamic-pituitary-adrenal axis. Endocrinology 150: 1931– 1944. 19095740 10.1210/en.2008-1471

[cow060C58] Stickney DG, Torres JJ (1989) Proximate composition and energy content of mesopelagic fishes from the eastern Gulf of Mexico. Mar Biol 103: 13– 24.

[cow060C59] Teal JM, Howarth RW (1984) Oil spill studies: a review of ecological effects. Environ Manage 8: 27–43.

[cow060C60] Velando A, Alvarez D, Mourino J, Arcos F, Barros A (2005) Population trends and reproductive success of the European shag *Phalacrocorax aristotelis* on the Iberian Peninsula following the Prestige oil spill. J Ornithol 146: 116–120.

[cow060C61] Walter ST, Carloss MR, Hess TJ, Leberg PL (2013) Hurricane, habitat degradation, and land loss effects on Brown Pelican nesting colonies. J Coastal Res 29: 187–195.

[cow060C62] Whitehead A (2013) Interactions between oil-spill pollutants and natural stressors can compound ecotoxicological effects. Integr Comp Biol 53: 635–647. 23842611 10.1093/icb/ict080 PMC3895973

[cow060C63] Wiens JA, Ford RG, Heinemann D (1984) Information needs and priorities for assessing the sensitivity of marine birds to oil spills. Biol Conserv 28: 21– 49.

[cow060C64] Wiens JA, Crist TO, Day RH, Murphy SM, Hayward GD (1996) Effects of the Exxon Valdez oil spill on marine bird communities in Prince William Sound, Alaska. Ecol Appl 6: 828–841.

[cow060C65] Wilkinson PM, Nesbitt SA, Parnell JF (1994) Recent history and status of the Eastern Brown Pelican. Wildlife Soc B 22: 420–430.

[cow060C66] Will A, Watanuki Y, Kikuchi DM, Sato N, Ito M, Callahan M, Wynne‐Edwards K, Hatch S, Elliott K, Slater L et al (2015) Feather corticosterone reveals stress associated with dietary changes in a breeding seabird. Ecol Evol 5: 4221–4232. 26664674 10.1002/ece3.1694 PMC4667832

[cow060C67] Williams CT, Kitaysky AS, Kettle AB, Buck CL (2008) Corticosterone levels of tufted puffins vary with breeding stage, body condition index, and reproductive performance. Gen Comp Endocrinol 158: 29–35. 18547575 10.1016/j.ygcen.2008.04.018

[cow060C68] Williams JC, Drummond BA, Buxton RT (2010) Initial effects of the August 2008 volcani eruption on breeding birds and marine mammals at Kasatochi Island, Alaska. Arct Antarct Alp Res 42: 306–314.

[cow060C69] Yaukey PH (2012) Population changes of urban land birds in the three years following the hurricane Katrina flood. Nat Hazards 61: 1203–1217.

[cow060C70] Yosef R, Gombobaatar S, Bortolotti GR (2013) Sibling competition induces stress independent of nutritional status in broods of Upland Buzzards. J Raptor Res 47: 127–132.

[cow060C71] Zimmer C, Boogert NJ, Spencer KA. 2013 Developmental programming: cumulative effects of increased pre-hatching corticosterone levels and post-hatching unpredictable food availability on physiology and behavior in adulthood. Horm Behav 64: 494–500. 23891687 10.1016/j.yhbeh.2013.07.002 PMC3791420

